# Wearable Device-Independent Next Day Activity and Next Night Sleep Prediction for Rehabilitation Populations

**DOI:** 10.1109/JTEHM.2020.3014564

**Published:** 2020-08-05

**Authors:** Allison Fellger, Gina Sprint, Douglas Weeks, Elena Crooks, Diane J. Cook

**Affiliations:** 1 Department of Computer ScienceGonzaga University 7447 SpokaneWA99258USA; 2 St. Luke’s Rehabilitation InstituteSpokaneWA99202USA; 3 Department of Physical TherapyEastern Washington University 2380 SpokaneWA99202USA; 4 School of Electrical Engineering and Computer ScienceWashington State University 6760 PullmanWA99164USA

**Keywords:** Actigraphy, activity and sleep prediction, inpatient rehabilitation, machine learning, wearable sensors

## Abstract

Wearable sensor-based devices are increasingly applied in free-living and clinical settings to collect fine-grained, objective data about activity and sleep behavior. The manufacturers of these devices provide proprietary software that labels the sensor data at specified time intervals with activity and sleep information. If the device wearer has a health condition affecting their movement, such as a stroke, these labels and their values can vary greatly from manufacturer to manufacturer. Consequently, generating outcome predictions based on data collected from patients attending inpatient rehabilitation wearing different sensor devices can be challenging, which hampers usefulness of these data for patient care decisions. In this article, we present a data-driven approach to combining datasets collected from different device manufacturers. With the ability to combine datasets, we merge data from three different device manufacturers to form a larger dataset of time series data collected from 44 patients receiving inpatient therapy services. To gain insights into the recovery process, we use this dataset to build models that predict a patient’s next day physical activity duration and next night sleep duration. Using our data-driven approach and the combined dataset, we obtained a normalized root mean square error prediction of 9.11% for daytime physical activity and 11.18% for nighttime sleep duration. Our sleep result is comparable to the accuracy we achieved using the manufacturer’s sleep labels (12.26%). Our device-independent predictions are suitable for both point-of-care and remote monitoring applications to provide information to clinicians for customizing therapy services and potentially decreasing recovery time.

## Graphical abstract

Previous daytime activity and nighttime sleep data collected from wearable sensors are used to predict a patient's next night sleep duration. A parameter, P, determines the number of previous periods to use for training a machine learning model to make the prediction.

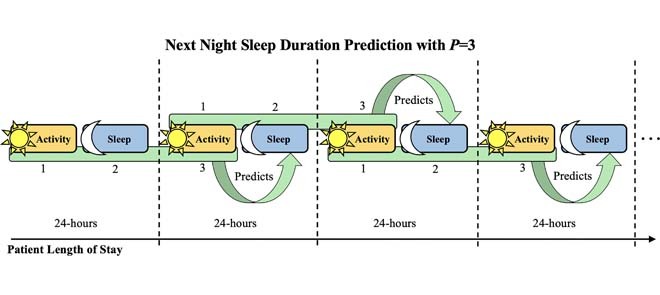

## I. Introduction

When an individual experiences an injury or illness that requires inpatient rehabilitation, the individual’s physical activity and sleeping patterns are often affected. Common reasons for undergoing inpatient rehabilitation include recovering from a traumatic brain injury (TBI), a stroke, cardiac disorders, lower extremity fractures, and various orthopedic surgeries. Specifically for patients with TBI, research has found that more than 66% of patients experience sleep disorders [1], while that number is as high as 78% for individuals recovering from a stroke [2]. Individuals recovering from TBI or stroke are often admitted to an inpatient rehabilitation facility to receive therapy services. Unfortunately, prescribed therapy may not be equally effective for each patient due to low levels of physical activity during the day and sleep disorders at night. Together, inactivity and sleep disorders can negatively impact the rest-activity circadian rhythm cycle that may slow the recovery from an injury or illness, even affecting quality of life [3]. Therefore, in inpatient rehabilitation, objective physical activity and sleep data can offer insights for clinicians to help customize therapy sessions with the goal of shortening the recovery process.

Objective data can be collected using wearable sensor-based devices that collect fine-grained physical activity and sleep data. Sensor-based physical activity and sleep measurements offer several benefits over human observation by therapists and by the patients themselves. Data collected from sensors remove the inaccuracy that is common amongst measurements that are self-reported by patients. For individuals with and without a health condition, it is difficult to objectively self-characterize activity and sleep. People tend to either overestimate or underestimate their activity, with correlations to direct measurement varying from −0.71 to 0.96 [4]. Secondly, data collected from sensors is not subject to variability due to inter-rater reliability. Sensor-based devices continuously track 24-hour physical activity and sleep in the same format and under the same conditions, allowing consistent data collection. Also, the technology has advanced enough to require minimal effort on the part of the clinician. This is primarily due to shorter device setup times and longer battery lives, permitting 24-hour recordings without the subjectivity that is frequently introduced by human observation.

Despite these advantages of sensor-based devices, their efficacy, accuracy, and applicability in inpatient rehabilitation settings remain areas of significant research. The majority of studies have focused on evaluating sensor-devices as well as their associated activity and sleep algorithms for healthy individuals. It is difficult to generalize such results to individuals with a health condition, such as those undergoing inpatient rehabilitation. Typically when these devices and their algorithms are applied to individuals with mobility impairments or sleep disorders, the results are highly variable [5] and can produce inexplicable results [6]. Additional challenges that arise from analyzing data collected from clinical settings include how to combine datasets from different sensor devices and how to use the combined data to help clinicians provide therapy services. There are several wearable sensor device manufacturers, and each one produces slightly different measurements of physical activity and sleep. While research has studied the validity of various manufacturers and their devices, the discrepancies across devices make it difficult for sleep researchers and clinicians to combine datasets and interpret the results [7].

To help alleviate this challenge and advance clinical activity and sleep research, we utilized research-grade Actigraph devices and consumer-grade pedometer devices in an inpatient rehabilitation facility to collect data from patients during their recovery process. Specifically, we utilized Ambulatory Monitoring Inc (AMI) MotionLogger devices, Philips Actiwatch Spectrum Plus devices, and Fitbit Charge devices with heart rate measurement capability. Using a data-driven approach, we combined data from these devices to implement a machine learning-based approach to measure and predict a patient’s future physical activity and sleep duration. Our results provide accurate predictions of activity levels for the forthcoming day and sleep duration for the forthcoming night. Our approach to manufacturer-independent physical activity and sleep prediction support point-of-care and remote patient monitoring that can help meet the needs of precision medicine by individualizing healthcare services [8].

## II. Related Work

Wearable sensor-based devices, like Actigraphs and Fitbits, are wrist-worn devices that are less obtrusive and less expensive alternatives to gold-standard methods. For sleep analysis, the commonly-used gold-standard technique for wearable sensor evaluation is polysomnography [9]. For physical activity, the gold-standard techniques include direct observation and motion capture systems [10]. At a minimum, wrist-worn devices typically contain tri-axial accelerometers that measure the acceleration of the wearer’s wrist for a short time interval, such as a second. Manufacturers of these devices process the acceleration time series data to determine a more clinically-relevant measure of physical activity than the original raw acceleration values, namely activity counts in the case of Actigraphs and step counts in the case of pedometers. When raw acceleration signals are combined with other sensor signals, such as heart rate or ambient light, algorithms can accurately label time intervals as “sleep” or “wake.” These labels are used by researchers and clinicians to determine if the wearer is sleeping or awake.

Manufacturers and researchers have investigated the error between gold-standard measurements and the output of manufacturer processing algorithms; however, this research has primarily used healthy subjects for evaluation [11]–[14]. While these algorithms perform well on healthy individuals, the algorithms can have higher error for individuals with a health condition, such as those recovering from an injury or illness like stroke or TBI, who exhibit highly irregular sleep and activity patterns [15]. To address this, recent research studying both healthy and unhealthy populations has focused on evaluating wearable sensor-based devices specifically for counting steps [10], detecting sleep periods [5], [16], and measuring sleep characteristics [17]–[20], such as total sleep time, sleep efficiency, number of awakenings, sleep onset latency, and wake after sleep onset.

We deployed three devices from different manufacturers for continuous data collection, so here we summarize the research investigating the accuracy of these specific devices. The three devices include MotionLogger, Actiwatch, and Fitbit. Beginning with physical activity measurements, for healthy adults in free-living conditions, a wrist-worn Fitbit has been shown to not differ significantly from a waist-worn ActiGraph GT3X for counting steps taken per minute over a 24-hour period [14]. For patients in a cardiac rehabilitation population, a wrist-worn Fitbit has been shown to correlate well with step count estimates from an Actigraph ($r=0.95$); however, the Fitbit tended to over-count steps [21]. When compared to direct observation, a wrist-worn Fitbit was reported to underestimate step count by 16% during a self-paced walking test performed by older adults with impaired ambulation [22]. In another study of subjects with multiple sclerosis performing a 2-min walk test, the Fitbit step count correlation ($r=0.69$) was lower than the Actigraph correlation ($r=0.76$) [23]. For the MotionLogger device, no clear difference between the accelerometer counts measured by the device and indirect calorimetry were detected during level walking [24].

For sleep measurements, strong correlations have been reported in healthy adults between estimates of total sleep time using a Philips Actiwatch and based on polysomnography ($r=0.94$), as well as betwee a wrist-worn Fitbit and polysomnography ($r=0.97$) [25]. In the same study, sleep efficiency measures from the Actiwatch and Fitbit did not differ from sleep efficiency measured by polysomnography. Therefore, total sleep time and sleep efficiency appear to be monitored by Actiwatch and Fitbit with reasonable accuracy. On the other hand, the MotionLogger device was found to underestimate total sleep time by almost 24 minutes and overestimate wake time by 25 minutes in healthy children and adolescents [26]. The same study found that the Philips Actiwatch did not demonstrate significant differences for total sleep time when controlling for age and sleep-disordered breathing.

These studies have found that though wearable devices do not demonstrate perfect measurements of activity and sleep for populations with health conditions, they do produce reasonable estimates. The next research step is to utilize these activity and sleep estimates to determine if they can help customize therapy for individual patients. One way to provide additional insights for customization is for an automated machine learning system to produce predictions about an individual’s future physical activity and sleep performance. Machine learning models generally benefit from being trained with large datasets. To acquire as much data as possible for human activity learning, several studies have investigated device-orientation independent methods for data collection [27], [28], fusing data from multiple sensors [29], and transfer learning approaches [30], [31].

Research using machine learning models for activity and sleep applications has primarily focused on classifying different types of physical activity [32], [33] and various sleep characteristics for healthy populations [11], [34], [35]. The work that is most similar to this article is that of Sathyanarayana and colleagues [11]. Sathyanarayana and colleagues collected Actigraph data from 92 healthy adolescents wearing ActiGraph GT3X+ devices for one week. Machine learning models trained with the collected daytime physical activity data were used to classify good and poor sleep efficiency with an area under the receiver operating curve of 0.9449. In our recent work, we expanded this research to investigate the applicability of sleep prediction for individuals with sleep disorders [36]. For this study, we deployed AMI MotionLogger devices in an inpatient rehabilitation setting. We continuously collected activity and sleep data from 17 inpatient rehabilitation subjects with identified sleep problems due to recovering from a stroke or TBI. Using this data, we constructed machine learning regression models to predict a patient’s future night sleep duration. Our regression approach achieved a 14.40% normalized root mean square error predicting next night sleep minutes.

## III. Methods and Procedures

In this article, we design approaches to data fusion and activity/sleep prediction. We then evaluate these approaches based on a sample of 44 subjects. Data were collected from subjects receiving inpatient therapy services for a variety of ailments, including stroke, TBI, cardiac disorders, pulmonary disorders, and lower extremity fractures. These subjects wore one of three different wearable-sensor devices: a MotionLogger, an Actiwatch, or a Fitbit. Because of these different devices, we apply a data-driven approach to support normalizing and combining the data from different manufacturers. We employ this combination of minute-by-minute activity and sleep data to make predictions about future nighttime sleep total inactive minutes (TIM) and total sleep time (TST) as measured by the device manufacturers. In addition to predicting a patient’s next night sleep duration, we also predict a patient’s next day total active minutes (TAM) to gain insight about daytime behavior.

### A. Data Collection

For data collection and analysis purposes, we define a 24-hour day as a period beginning at 06:00:00 and ending at 05:59:00 the following calendar day. Using known controlled lighting times at the inpatient facility, we determined the daytime (DT) period to coincide with when the lights were typically on in patients’ rooms, which was from 06:00:00 to 20:59:00. The nighttime (NT) period corresponded to the time period when lights were off in patients’ rooms, which was from 21:00:00 to 05:59:00. For ease of explanation, we denote successive NT and DT periods using the $\gg $ symbol. For example, the notation NT $\gg $ DT $\gg $ NT describes a nighttime, then daytime, then the following nighttime sequence which represents 9 + 15 + 9 = 33 continuous hours. We identify a period in a sequence of successive DT and NT periods using a subscript, such as DT$_{1} \gg $ NT$_{1} \gg $ DT_2_, where DT_1_ and NT_1_ are sampled from the same 24-hour period, and DT_2_ is from the next period.

We deployed and collected data from three different wearable sensor-based devices. These three devices represent three datasets we collectively analyze in this article to evaluate our prediction approach. The three datasets and their devices, with sample sizes, are as follows:

1) The “AMI” dataset: Ambulatory Monitoring Inc Basic MotionLogger Actigraph devices (N = 17)
2) The “Philips” dataset: Philips Actiwatch Spectrum Plus Actigraph devices (N = 19)
3) The “Fitbit” dataset: Fitbit Charge with Heart Rate pedometer devices (N = 8)

**FIGURE 1.**
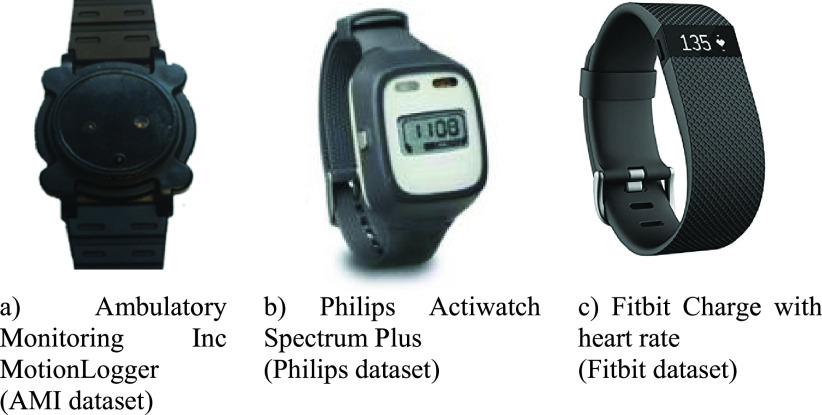
The three devices used for data collection.

For reference, Figure 1 includes images of these devices. In total, we collected continuous data from 44 patients undergoing inpatient rehabilitation. Patients admitted to the hospital following an injury or illness, such as stroke or TBI, were recruited to participate in these studies if their therapist stated they were experiencing irregular sleeping patterns. The data collection protocols for all three datasets were approved by our local institutional review board and all patients provided written informed consent to participate. Each subject continuously wore one of these three devices during both the daytime and nighttime periods until they were discharged from the rehabilitation facility.

For the AMI dataset, we analyzed data from 17 subjects (age 64.11 ± 17.05 years; 11 females and 6 males), for which the data collection periods ranged from 9 days to 30 days [36]. For the Philips dataset, we analyzed data from 22 subjects (age 63.96 ± 17.93 years; 5 females and 17 males), for which the data collection periods ranged from 4 days to 30 days. For the AMI and Philips datasets, the device manufacturers provided Actigraph-style activity counts and binary “sleep” or “wake” labels for each minute of data collection. We used these activity counts to represent physical activity.

For the Fitbit dataset, we originally collected data from 15 subjects who participated in the study during the duration of their inpatient rehabilitation stay [6], [37]. For several of these 15 subjects, there were entire nights with missing sleep data, likely due to patients taking the device off and/or the Fitbit sleep algorithms not properly detecting the wearer’s abnormal sleeping patterns. Therefore, in this study we only used the data collected from eight participants for whom high-integrity sleep data were available every night of data collection (age 66.25 ± 12.89 years; 6 females and 2 males), for which the data collection periods ranged from 5 days to 17 days. Instead of recording activity counts, Fitbit labels each minute with a number of “steps” taken. We used steps as a similar measure to the aforementioned activity counts to estimate a subject’s physical activity. For labeling sleep, Fitbit provides four levels of sleep: 0 (no sleep) or 1, 2, 3, (increasing levels of deeper sleep). To align this data with that of the AMI and Philips datasets, we reclassified these four sleep levels into binary sleep/wake labels where a value of 0 was re-labeled as “wake” and a value of 1, 2, or 3 was re-labeled as “sleep.”. In summary, across all three datasets there was a total of 596 days of data collected in this study.

### B. Data Preprocessing

The three datasets consisted of minute-by-minute physical activity and sleep/wake time series data. We preprocessed these time series to prepare the data for consistent analyses across the different wearable device manufacturers. We framed each subject’s time series data to start on the first day with at least 400 consecutive minutes of recorded activity and to end on the day with at least 800 consecutive minutes of no recorded activity. We then normalized each subject’s activity counts (AMI and Philips datasets) or steps (Fitbit dataset) to be between 0 and 1.

**FIGURE 2.**
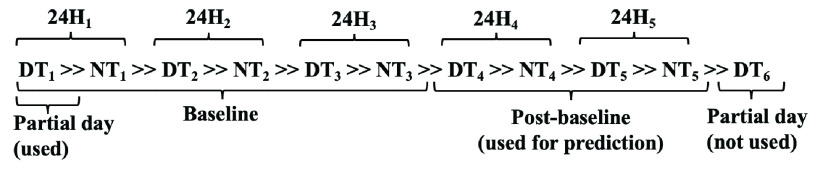
Example extracting daytime (DT) and nighttime (NT) periods into baseline and post-baseline for a subject with five 24-hour (24H) days of data.

Because the three datasets were each sampled from different devices, we computed our own normalized labels for each individual subject. For each subject, we provided an “active” or “inactive” label for each minute in the time series data. These labels represent platform-independent labels that offer an alternative to individual device manufacturer’s activity and sleep labels. To assign our individualized minute labels, we held out the first three days of data collection for each subject to serve as a baseline period. Using a subject’s own data as a multiple-day baseline allowed us to account for extreme variability across subjects’ data. We divided the baseline data into DT and NT periods, for a total of three baseline DT periods and three baseline NT periods. Figure 2 provides an example of how the baseline period was extracted from an example subject with five days of data collection. From these baseline periods, we extracted baseline activity means (BAM), namely, the DT baseline activity mean DT_BAM_ and the NT activity mean NT_BAM_. We decided to use the mean of baseline activity because it was highly correlated with manufacturer sleep and wake labels (see Section IV for results). Using the DT_BAM_ for each subject, we labeled the remaining post-baseline DT minutes for the subject as active if its activity value was greater than DT_BAM_, or inactive if it was less or equal to DT_BAM_. We repeated this process for the NT periods, using NT_BAM_. Our BAM labeling algorithm provided subject-specific and device-independent labels for daytime activity and nighttime activity. For nighttime activity, we hypothesized that the inactive labels were indicative of sleep and we evaluated this hypothesis by comparing the inactive labels to the manufacturer-provided sleep/wake labels.

### C. Feature Extraction

From the time series data for each subject, we extracted relevant physical activity and sleep quality features from the DT and NT periods separately. To determine physical activity during DT periods, we used both the manufacturer activity counts and our aforementioned BAM labels. For each 24-hour day, we counted the number of BAM-labeled DT active minutes, as well as the number of transitions from active to inactive. We also computed a daytime activity ratio, which is the daytime sum of the manufacturer-measured activity divided by the corresponding 24-hour total.

We computed NT features using both the minute-by-minute manufacturer’s sleep/wake time series and our BAM-labeled inactive minutes. We extracted nighttime TST, number of sleep transitions, sleep onset latency (number of minutes from the start of nighttime before sleep), longest sleep bout length, and wake after sleep onset [17]. In addition to daytime and nighttime features, we included the number of days since each subject’s injury or illness as a feature. To summarize, Table 1 lists the labels and features that were used for predicting DT TAM, NT TIM, and NT TST, respectively.

**TABLE 1:** Predicted Values and Their Labels Used for Feature Extraction

Predicted Value	DT Feature Labels	NT Feature Labels
DT TAM	BAM active mins	BAM inactive mins
NT TIM	BAM active mins	BAM inactive mins
NT TST	BAM active mins	Manufacturer sleep/wake mins

BAM=baseline activity means, TAM=total active minutes, TIM=total inactive minutes, TST=total sleep time labeled by the manufacturer.

**FIGURE 3.**
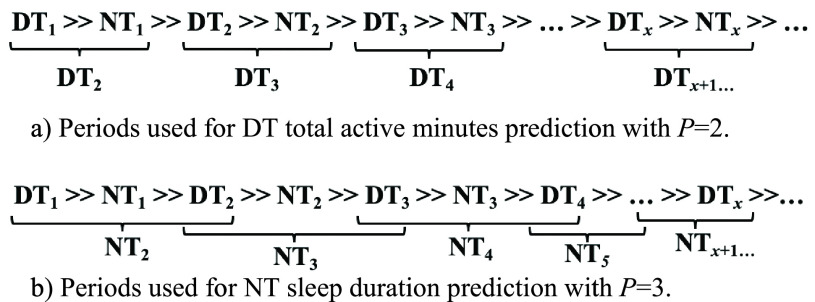
Example of daytime (DT) and nighttime (NT) periods used for DT total active minutes prediction (a) and used for sleep duration prediction (b) with example $P$ values.

We extracted the aforementioned features from a sequence of $P$ number of DT and NT periods to predict TAM for the following DT period, TIM for the following NT period, or TST for the following NT period. For example, if $P=1$ and we are predicting TST, then we use the manufacturer’s sleep/wake features from $P$-sequence DT_1_ to predict NT_1_, DT_2_ to predict NT_2_, and so forth. If $P=3$, then we use the $P$-sequence DT$_{1} \gg $ NT$_{1} \gg $ DT_2_ to predict NT_2_, DT$_{2}~\gg $ NT$_{2} \gg $ DT_3_ to predict NT_3_, and so forth (see Figure 3 for a diagram showing both DT and NT predictions with different $P$ values). Because we did not include the three-day baseline for prediction and there was a subject in the Fitbit dataset with only five days of data collection, the maximum $P$ value was $P=2$ for DT TAM predictions and $P=3$ for NT TST predictions. Additionally, excluding the three-day baseline reduced the total combined dataset size to 464 days.

### D. Prediction Models

To predict TAM, TIM, and TST based on the extracted features, we utilized a 100-tree random forest, employing a mean squared error feature-selection criterion, enhanced with bagging, and without a maximum depth value [38]. We chose a random forest regression algorithm because it is an ensemble approach that exhibited low variance and yielded high prediction accuracy in our previous work predicting nighttime sleep duration [36]. Furthermore, the random forest regressor outperformed other regression models we explored, including $K$-nearest neighbors regressors, support vector regressors, and neural networks. We trained the random forest regressor using a fixed random seed of zero for reproducible results and evaluated the performance using leave-one-out cross-validation. For leave-one-out-cross-validation, each of the participant periods was held out as a test sample while the remaining $P$-sequences were used for training. Our initial nighttime predictions started on the first night following the three-day baseline. We excluded $P$-sequences if they represented future data from the same participant as the test data. For example, when $P=1$, there were 464 total NT periods, from which we held out one nighttime period, NT_*x*_ for leave-one-out cross-validation. We then excluded all periods $> x$ from training that were collected from the same participant as NT_*x*_.

To improve our random forest prediction accuracy, we utilized a $K$-nearest neighbors ($K$NN) algorithm to select $K$ “similar” $P$-sequences from the training set. The $K$ smallest distances between a test $P$-sequence and all other $P$-sequences in a subgroup were selected to form a smaller, more specialized training set. Two $P$-sequences were considered similar if they were in the same subgroup and yielded a small Euclidean distance between their feature vectors. We investigated alternatives to distance calculation, including using dynamic time warping to compare two $P$-sequences. Experiments revealed the best results were achievable using feature vector-based Euclidean distance. The subgroup parameter restricted which feature vectors were considered similar to the held-out feature vector. Subgroups we explored included dataset, gender, and no subgroupings (using all $P$-sequences). With the $K$ parameter we aimed to train on a minimal set of $P$-sequences that historically were similar to the current $P$-sequence for which we were making a prediction.

## IV. Results

To evaluate our BAM data-driven approach to normalizing data collected from different devices, we correlated the original manufacturer-provided sleep/wake time series with our BAM-labeled time series. We experimented with various threshold values for determining active/inactive states, including the baseline mean and percentiles in increments of ten. Table 2 shows the top three correlations for the AMI and Philips datasets. For the Fitbit dataset, the correlations for mean and all percentiles tested were the same, $r=0.53$. This is because Fitbit typically measures zero steps when the wearer is laying down and does not exhibit wrist motion similar to an arm swing during a step. This means that during NT periods, there were almost exclusively zero values, causing any minute with more than zero steps to be labeled as active.

**TABLE 2:** Correlations Between Nighttime Manufacturer Sleep/Wake Values and BAM Inactive Values

Dataset	Activity Metric	Threshold	Correlation
AMI	Activity count	Mean	0.78
	Activity count	70^th^	0.72
	Activity count	80^th^	0.68
Philips	Activity count	Mean	0.59
	Activity count	70^th^	0.56
	Activity count	80^th^	0.55
Fitbit	Steps	All	0.53

All correlations were significant at p<0.01. Correlations for all Fitbit dataset thresholds tested were the same value.

To standardize features for input to machine learning models, we subtracted the mean and scaled to unit variance. Next, we trained and tested the prediction models using leave-one-out-cross-validation. We evaluated the random forest regression results using mean absolute error (MAE), root mean squared error (RMSE), normalized RMSE (NRMSE), and Pearson correlation coefficients ($r$). We computed MAE as the sum of the absolute values of the difference between the predicted values and the actual ground truth values divided by the number of predictions, as show in Equation 1:

\begin{equation*} \mathrm {MAE=}\frac {\sum \nolimits _{\mathrm {i=1}}^{\mathrm {n}} \left |{ {\mathrm {prediction}}_{\mathrm {i}}-{\mathrm {actual}}_{\mathrm {i}} }\right |}{\mathrm {n}}\tag{1}\end{equation*}

We computed RMSE as the square root of the sum of the squares of the difference between the predicted values and the actual ground truth values divided by the number of predictions, as show in Equation 2:

\begin{equation*} \mathrm {RMSE=}\sqrt {\frac {\sum \nolimits _{\mathrm {i=1}}^{\mathrm {n}} \left ({{\mathrm {prediction}}_{\mathrm {i}}-{\mathrm {actual}}_{\mathrm {i}} }\right)^{2}}{\mathrm {n}}}\tag{2}\end{equation*}

We computed NRMSE as RMSE divided by the difference between the maximum and minimum actual ground truth values, as shown in Equation 3:

\begin{equation*} \mathrm {NRMSE=}\frac {\mathrm {RMSE}}{\mathrm {actua}\mathrm {l}_{\mathrm {max}}\mathrm {-actua}\mathrm {l}_{\mathrm {min}}}\tag{3}\end{equation*}

Lastly, we computed the correlation coefficients as the Pearson correlation and its associated p-value calculated between the predicted values and the actual ground truth values.

**FIGURE 4.**
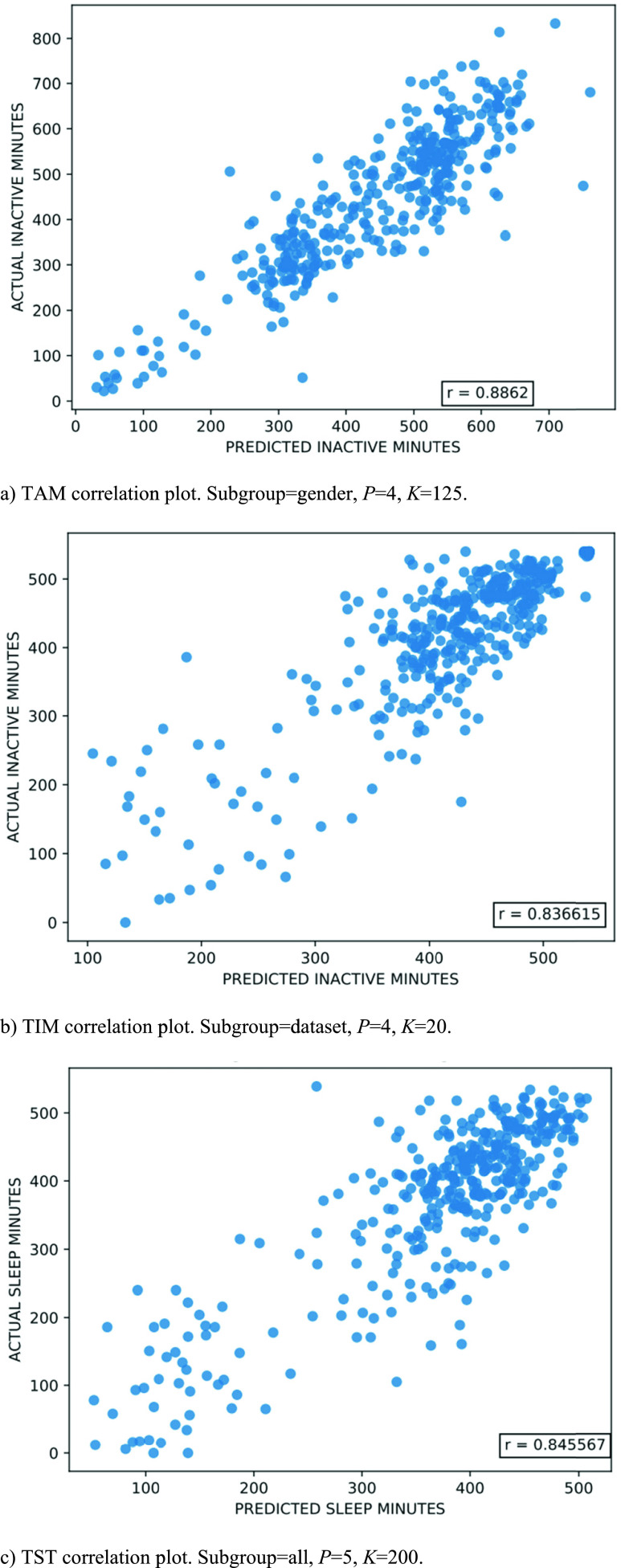
Correlation plots showing top regression results for total active minutes (TAM), total inactive minutes (TIM), and total sleep time (TST) actual versus predicted values.

Tables 3–5 summarize five random forest results and their parameter configurations, in ascending NRMSE order, for DT TAM, NT TIM, and NT TST, respectively. To provide context for interpreting the prediction results, DT TAM, demonstrated a mean and standard deviation of 431.20 ± 162.45 minutes (coefficient of variation equal to 37.67%) across the participant group, while the NT TIM and TST had a mean and standard deviation of 414.22 ± 108.61 minutes (coefficient of variation equal to 26.22%) and 370.27 ± 124.20 minutes (coefficient of variation equal to 33.54%), respectively. To more thoroughly investigate the prediction results, we provide scatter plots of predicted total minutes versus actual total minutes for the best TAM (Figure 4a), TIM (Figure 4b), and TST (Figure 4c) results from Tables 3–5. For each of the figures, the data correlation is indicated as an annotation in the bottom right corner of the plot.

**TABLE 3:** Best Daytime Total Active Minutes (TAM) Prediction Results

Subgroup	$P$	$K$	MAE	RMSE	NRMSE	$r$
Gender	4	125	55.73	73.92	9.11%	0.89
All	4	150	55.45	73.96	9.12%	0.89
All	4	250	56.04	74.54	9.19%	0.88
Gender	4	50	57.18	74.65	9.20%	0.88
Gender	4	75	57.04	74.67	9.21%	0.88

MAE=mean absolute error, RMSE=root mean squared error,

NRMSE=normalized RMSE, and $r=$Pearson correlation coefficient. All correlation results were statistically significant at p<0.0001.

**TABLE 4:** Best Nighttime Total Inactive Minutes (TIM) Prediction Results

Subgroup	$P$	$K$	MAE	RMSE	NRMSE	$r$
Dataset	4	20	43.61	60.37	11.18%	0.84
Dataset	4	15	43.70	60.85	11.27%	0.83
Dataset	4	10	43.22	61.37	11.37%	0.83
Dataset	5	10	44.45	62.16	11.51%	0.83
All	4	300	46.17	63.63	11.78%	0.82

MAE=mean absolute error, RMSE=root mean squared error,

NRMSE=normalized RMSE, and $r=$Pearson correlation coefficient. All correlation results were statistically significant at p<0.0001.

**TABLE 5:** Best Nighttime Total Sleep Time (TST) Prediction Results

Subgroup	$P$	$K$	MAE	RMSE	NRMSE	$r$
All	5	200	50.56	66.07	12.26%	0.85
All	4	200	50.37	66.22	12.29%	0.84
All	5	150	50.52	66.41	12.32%	0.84
All	4	150	51.12	66.53	12.34%	0.84
All	4	300	51.06	66.86	12.40%	0.84

MAE=mean absolute error, RMSE=root mean squared error,

NRMSE=normalized RMSE, and $r=$Pearson correlation coefficient. All correlation results were statistically significant at p<0.0001.

**FIGURE 5.**
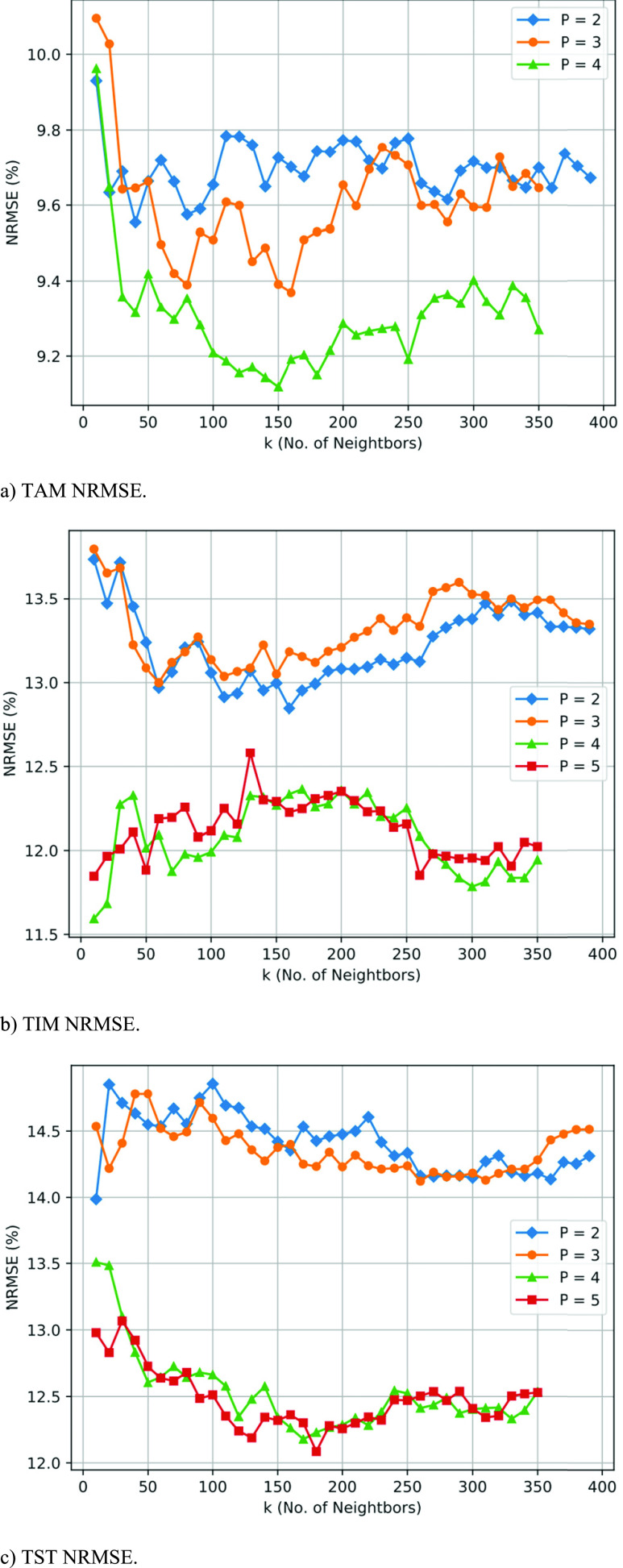
Normalized root mean square error (NRMSE) as a function of $K$ (number of similar $P$ sequences used for training) for different values of $P$. Plots for total active minutes (TAM), total inactive minutes (TIM), and total sleep time (TST) are shown.

We ran several experiments to explore the effects of parameter choices, including $P$ (the number of periods preceding the NT period) and $K$ (the number of similar $P$-sequences used by $K$NN to determine the training set). Figure 5 shows NRMSE values for alternative values of $P$ as a function of alternative values of $K$. $P=1$ exhibited relatively large error compared to the other $P$ values so we exclude it from the plots of Figure 5 to clearly illustrate the patterns of the lower $P$-value NRMSE results.

## V. Discussion

In this article, we propose the BAM data-driven approach for predicting daytime total active minutes and nighttime total inactive minutes for patients undergoing inpatient rehabilitation. Our approach allows data collected from different wearable device manufacturers to be combined, compared, and used for prediction. Of the different mean and percentiles from the three-day baseline period we explored as active minute thresholds, the baseline means exhibited the highest correlation to the manufacturer’s sleep and wake labels (see Table 2; AMI dataset $r=0.78$; Philips dataset $r=0.59$). Since the correlations were < 1.0, we anticipated the TIM prediction results using the BAM labels would be less accurate than the TST prediction results that used the manufacturer’s sleep/wake labels. The random forest results indicate that the top TAM and TST results are comparable (see Tables 4 and 5; TIM NRMSE 11.18%; TAMNRMSE 12.26%). This suggests that our BAM approach could be used not only for prediction, but also for building larger training sets with normalized sleep labels and for comparisons between subject’s data collected from different devices.

When training the random forest regressors on each dataset individually, the results are not as strong as the combined dataset. For the AMI dataset, the best NRMSE result for TIM is 14.11% and for TST is 14.98%. For the Philips dataset, these numbers are 11.45% for TIM and 13.12% for TST. For the Fitbit dataset, these numbers are 18.05% for TIM and 21.23% for TST. A likely reason that the Fitbit-based prediction is weaker than the other datasets is the small sample size of the Fitbit dataset (N = 8) and low detection of nighttime active minutes. The low nighttime active minutes is due to Fitbit measuring sedentary behavior using step counts instead of activity counts like Actigraphy-based devices. Since steps are less granular and capture a narrower range of physical activity, Actigraphy-based devices are more appropriate for tracking activity in inpatient rehabilitation patients who exhibit a wide range of physical activity. This observation further supports the idea of pooling data together to form larger datasets, which would provide more similar $P$-sequences detected with $K$NN and used for training.

For the TAM results, there are no manufacturer “active” or “inactive” labels with which to compare our BAM approach; however, we do see lower prediction error for TAM (see Table 3; 9.11% NRMSE and $r=0.89$). Breaking this result down by dataset reveals that, like the NT period predictions, the prediction results vary by device and are strongest when pooled together. The AMI dataset’s best TAM NRMSE is 13.96%, while that number is 10.48% for the Philips dataset, and 11.48% for the Fitbit dataset. These NT and DT prediction results further suggest the importance of combining datasets to increase training set size and consequently prediction accuracy. To deploy models in a clinical setting, such steps are needed to achieve the lowest possible error.

To more thoroughly explore the 9-12% error rates, we include correlation plots in Figure 4. The prediction results align with our intuition. The random forest regressors are more accurate when the actual minutes being predicted are closer to the mean, which is the case for the NT periods. In Figures 4b and 4c, there is a cluster of high actual total inactive minutes and sleep minutes. With a larger, more diverse dataset, we anticipate custom models could be built for the outlier subjects with lower active and inactive minutes to improve accuracy.

Next we explore the effects of the $P$ and $K$ parameters on the TAM, TIM, and TST prediction accuracies. Investigating the plots in Figure 5, we observe large benefits to increasing $P$ from 1 to 2, from 2 to 3, and from 3 to 4. The NRMSE curves for $P=4$ and $P=5$ are fairly similar, suggesting that accurate predictions can be made with as few as four post-baseline training periods. This constitutes a 48-hour period for a TAM prediction and a 63-hour period for a TIM prediction. We plan to investigate techniques to shorten this overall time from when a device is first worn by a patient to when an accurate next period prediction can be made. A few approaches include trimming periods from the baseline and constructing more individualized models for specific injuries or illnesses, such as stroke or TBI. The tradeoffs for the $K$ parameter are not as clear as for the $P$ parameter. $K$ appears to be more sensitive to which value is being predicted. For TAM, $K=150$ seems to capture the majority of the prediction improvement. For TIM, $K=50$ and for TST, $K=150$ are reasonable minimum values for achieving accurate results. Since larger values of $K$ do not greatly improve prediction accuracy, as datasets get larger, computational overhead from exploring optimal $K$ values is not expected to increase. However, the search space for determining the $K$ nearest neighbors will grow. Individualized models for different patient subgroups could also help limit the number of required $K$NN comparisons.

## VI. Conclusion

We investigated applying device-independent physical activity and sleep labels determined from a baseline period to allow data collected from multiple wearable devices to be combined into larger datasets. Larger datasets can be used to train machine learning models to predict a patient’s next day physical activity and next night sleep duration with greater accuracy. We demonstrated such prediction models trained with data collected from 44 inpatient rehabilitation subjects can achieve NRMSE values near 9% for daytime physical activity prediction and near 11% for nighttime sleep duration prediction. These results were an expansion over own prior work with data from a single sensor device [36]. For future work, we plan to continue growing our sample size to provide additional historical data sequences for $K$NN to select from. We anticipate this will help prediction accuracy for outlier $P$-sequences sampled from subjects with highly irregular activity and sleep behavior. We also plan to apply deep learning models [11] in an effort to further reduce the nighttime prediction error. Our eventual goal is to deploy models that are accurate enough for clinicians to use to help customize individual patient therapy programs. If such a system can make accurate predictions in near real time, clinicians could use this additional information about a patient’s next day physical activity and next night sleep requirements to adapt forthcoming therapeutic activities and potentially shorten the recovery process.

## References

[1] Mathias J. L. and Alvaro P. K., “Prevalence of sleep disturbances, disorders, and problems following traumatic brain injury: A meta-analysis,” Sleep Med., vol. 13, no. 7, pp. 898–905, Aug. 2012. 22705246 10.1016/j.sleep.2012.04.006

[2] Pasic Z., Smajlovic D., Dostovic Z., Kojic B., and Selmanovic S., “Incidence and types of sleep disorders in patients with stroke,” Med. Arch., vol. 65, no. 4, p. 225, 2011. 10.5455/medarh.2011.65.225-227 21950229

[3] Innominato P. F., “Circadian rhythm in rest and activity: A biological correlate of quality of life and a predictor of survival in patients with metastatic colorectal cancer,” Cancer Res., vol. 69, no. 11, pp. 4700–4707, Jun. 2009. 19470769 10.1158/0008-5472.CAN-08-4747 PMC2690615

[4] Prince S. A., Adamo K. B., Hamel M., Hardt J., Connor Gorber S., and Tremblay M., “A comparison of direct versus self-report measures for assessing physical activity in adults: A systematic review,” Int. J. Behav. Nutrition Phys. Activity, vol. 5, no. 1, p. 56, 2008. 10.1186/1479-5868-5-56 PMC2588639 18990237

[5] Sadeh A., “The role and validity of actigraphy in sleep medicine: An update,” Sleep Med. Rev., vol. 15, no. 4, pp. 259–267, Aug. 2011. 21237680 10.1016/j.smrv.2010.10.001

[6] Sprint G., Cook D., Weeks D., Dahmen J., and La Fleur A., “Analyzing sensor-based time series data to track changes in physical activity during inpatient rehabilitation,” Sensors, vol. 17, no. 10, p. 2219, Sep. 2017. 10.3390/s17102219 PMC5677114 28953257

[7] Berger A. M., Wielgus K. K., Young-McCaughan S., Fischer P., Farr L., and Lee K. A., “Methodological challenges when using actigraphy in research,” J. Pain Symptom Manage., vol. 36, no. 2, pp. 191–199, Aug. 2008. 18400460 10.1016/j.jpainsymman.2007.10.008 PMC2542506

[8] Bigelow M. E. G., “Point-of-care technologies for the advancement of precision medicine in heart, lung, blood, and sleep disorders,” IEEE J. Translational Eng. Health Med., vol. 4, pp. 1–10, 2016. 10.1109/JTEHM.2016.2593920 PMC5003165 27602308

[9] de Souza L., Benedito-Silva A. A., Pires M. L. N., Poyares D., Tufik S., and Calil H. M., “Further validation of actigraphy for sleep studies,” Sleep, vol. 26, no. 1, pp. 81–85, Jan. 2003. 12627737 10.1093/sleep/26.1.81

[10] Treacy D., Hassett L., Schurr K., Chagpar S., Paul S. S., and Sherrington C., “Validity of different activity monitors to count steps in an inpatient rehabilitation setting,” Phys. Therapy, vol. 97, no. 5, pp. 581–588, 5 2017. 10.1093/ptj/pzx010 28339904

[11] Sathyanarayana A., “Sleep quality prediction from wearable data using deep learning,” JMIR mHealth uHealth, vol. 4, no. 4, p. e125, Nov. 2016. 27815231 10.2196/mhealth.6562 PMC5116102

[12] Ohayon M. M., Carskadon M. A., Guilleminault C., and Vitiello M. V., “Meta-analysis of quantitative sleep parameters from childhood to old age in healthy individuals: Developing normative sleep values across the human lifespan,” Sleep, vol. 27, no. 7, pp. 1255–1273, Oct. 2004. 15586779 10.1093/sleep/27.7.1255

[13] Alinia P., Cain C., Fallahzadeh R., Shahrokni A., Cook D., and Ghasemzadeh H., “How accurate is your activity tracker? A comparative study of step counts in low-intensity physical activities,” JMIR mHealth uHealth, vol. 5, no. 8, p. e106, Aug. 2017. 28801304 10.2196/mhealth.6321 PMC5572056

[14] Dominick G. M., Winfree K. N., Pohlig R. T., and Papas M. A., “Physical activity assessment between consumer- and research-grade accelerometers: A comparative study in free-living conditions,” JMIR mHealth uHealth, vol. 4, no. 3, p. e110, Sep. 2016. 27644334 10.2196/mhealth.6281 PMC5048058

[15] Roomkham S., Lovell D., Cheung J., and Perrin D., “Promises and challenges in the use of consumer-grade devices for sleep monitoring,” IEEE Rev. Biomed. Eng., vol. 11, pp. 53–67, 2018. 29993607 10.1109/RBME.2018.2811735

[16] Makley M. J., “Objective measures of sleep and wakefulness in patients with moderate to severe brain injury on an inpatient rehabilitation unit. Pearls and pitfalls of actigraph monitoring,” NeuroRehabilitation, vol. 43, no. 3, pp. 277–285, Nov. 2018. 30373965 10.3233/NRE-182537

[17] Mendonca F., Mostafa S. S., Morgado-Dias F., Ravelo-Garcia A. G., and Penzel T., “A review of approaches for sleep quality analysis,” IEEE Access, vol. 7, pp. 24527–24546, 2019.

[18] Chiu H.-Y., Chen P.-Y., Chen N.-H., Chuang L.-P., and Tsai P.-S., “Trajectories of sleep changes during the acute phase of traumatic brain injury: A 7-day actigraphy study,” J. Formosan Med. Assoc., vol. 112, no. 9, pp. 545–553, Sep. 2013. 23906685 10.1016/j.jfma.2013.06.007

[19] Leger D., “Using actigraphy to assess sleep and wake rhythms of narcolepsy type 1 patients: A comparison with primary insomniacs and healthy controls,” Sleep Med., vol. 52, pp. 88–91, Dec. 2018. 30286385 10.1016/j.sleep.2018.07.024

[20] Sainath R. V. S. N. Y., Pruthvisai K., Akhil B. V. H. A. R., and Palaniswamy S., “Sleep pattern monitoring and analysis to improve the health and quality of life of people,” in Proc. Int. Conf. Adv. Comput., Commun. Informat. (ICACCI), Sep. 2018, pp. 1900–1905.

[21] Alharbi M., Bauman A., Neubeck L., and Gallagher R., “Validation of fitbit-flex as a measure of free-living physical activity in a community-based phase III cardiac rehabilitation population,” Eur. J. Preventive Cardiol., vol. 23, no. 14, pp. 1476–1485, Sep. 2016. 10.1177/2047487316634883 26907794

[22] Floegel T. A., Florez-Pregonero A., Hekler E. B., and Buman M. P., “Validation of consumer-based hip and wrist activity monitors in older adults with varied ambulatory abilities,” Journals Gerontol. Ser. A, Biol. Sci. Med. Sci., vol. 72, no. 2, pp. 229–236, Feb. 2017. 10.1093/gerona/glw098 PMC6082588 27257217

[23] Block V. J., “Continuous daily assessment of multiple sclerosis disability using remote step count monitoring,” J. Neurol., vol. 264, no. 2, pp. 316–326, Feb. 2017. 27896433 10.1007/s00415-016-8334-6 PMC5292081

[24] Westerterp K., “Physical activity assessment with accelerometers,” Int. J. Obesity, vol. 23, no. S3, pp. S45–S49, Apr. 1999. 10.1038/sj.ijo.0800883 10368002

[25] Mantua J., Gravel N., and Spencer R., “Reliability of sleep measures from four personal health monitoring devices compared to research-based actigraphy and polysomnography,” Sensors, vol. 16, no. 5, p. 646, 5 2016. 10.3390/s16050646 PMC4883337 27164110

[26] Meltzer L. J., Walsh C. M., Traylor J., and Westin A. M. L., “Direct comparison of two new actigraphs and polysomnography in children and adolescents,” Sleep, pp. 159–166, Jan. 2012. 22215930 10.5665/sleep.1608 PMC3242684

[27] Thiemjarus S., “A device-orientation independent method for activity recognition,” in Proc. Int. Conf. Body Sensor Netw., Jun. 2010, pp. 19–23.

[28] Wu J. and Jafari R., “Orientation independent activity/gesture recognition using wearable motion sensors,” IEEE Internet Things J., vol. 6, no. 2, pp. 1427–1437, Apr. 2019.

[29] Skubic M., Guevara R. D., and Rantz M., “Automated health alerts using in-home sensor data for embedded health assessment,” IEEE J. Translational Eng. Health Med., vol. 3, pp. 1–11, 2015. 10.1109/JTEHM.2015.2421499 PMC4848095 27170900

[30] Saeedi R. and Gebremedhin A. H., “A signal-level transfer learning framework for autonomous reconfiguration of wearable systems,” IEEE Trans. Mobile Comput., vol. 19, no. 3, pp. 513–527, Mar. 2020.

[31] Cook D., Feuz K. D., and Krishnan N. C., “Transfer learning for activity recognition: A survey,” Knowl. Inf. Syst., vol. 36, no. 3, pp. 537–556, Sep. 2013. 24039326 10.1007/s10115-013-0665-3 PMC3768027

[32] Lee K. and Kwan M.-P., “Physical activity classification in free-living conditions using smartphone accelerometer data and exploration of predicted results,” Comput., Environ. Urban Syst., vol. 67, pp. 124–131, Jan. 2018.

[33] Ellis K., Kerr J., Godbole S., Lanckriet G., Wing D., and Marshall S., “A random forest classifier for the prediction of energy expenditure and type of physical activity from wrist and hip accelerometers,” Physiol. Meas., vol. 35, no. 11, pp. 2191–2203, Oct. 2014. 25340969 10.1088/0967-3334/35/11/2191 PMC4374571

[34] Hidayat W., Tambunan T. D., and Budiawan R., “Empowering wearable sensor generated data to predict changes in Individual’s sleep quality,” in Proc. 6th Int. Conf. Inf. Commun. Technol. (ICoICT), May 2018, pp. 447–452.

[35] Farajtabar M., Kiciman E., Nathan G., and White R. W., “Modeling behaviors and lifestyle with online and social data for predicting and analyzing sleep and exercise quality,” Int. J. Data Sci. Analytics, vol. 8, no. 4, pp. 367–383, Jun. 2018.

[36] Fellger A., Sprint G., Andrews A., Weeks D., and Crooks E., “Nighttime sleep duration prediction for inpatient rehabilitation using similar actigraphy sequences,” in Proc. IEEE Healthcare Innov. Point Care Technol. (HI-POCT), Nov. 2019, pp. 41–44.

[37] Conci J., Sprint G., Cook D., and Weeks D., “Utilizing consumer-grade wearable sensors for unobtrusive rehabilitation outcome prediction,” in Proc. IEEE EMBS Int. Conf. Biomed. Health Informat. (BHI), May 2019, pp. 1–4.

[38] Pedregosa F., “Scikit-learn: Machine learning in Python,” J. Mach. Learn. Res., vol. 12, pp. 2825–2830, Oct. 2011.

